# Apple-like Shape of Freezing Paraffin Wax Droplets and Its Origin

**DOI:** 10.3390/ma16165514

**Published:** 2023-08-08

**Authors:** Pritam Kumar Roy, Shraga Shoval, Nir Shvalb, Leonid A. Dombrovsky, Oleg Gendelman, Edward Bormashenko

**Affiliations:** 1 Chemical Engineering Department, Engineering Faculty, Ariel University, P.O. Box 3, Ariel 407000, Israel; pritamr256@gmail.com (P.K.R.); ldombr@yandex.ru (L.A.D.); 2 Department of Industrial Engineering and Management, Engineering Faculty, Ariel University, P.O. Box 3, Ariel 407000, Israel; 3 Department of Mechanical Engineering & Mechatronics, Faculty of Engineering, Ariel University, P.O. Box 3, Ariel 407000, Israel; 4 Heat Transfer Department, Joint Institute for High Temperatures, Moscow 111116, Russia; 5 X-BIO Institute, University of Tyumen, Tyumen 625003, Russia; 6 Faculty of Mechanical Engineering, Technion-Israel Institute of Technology, Haifa 3200003, Israel

**Keywords:** droplet, dimple, shape, freezing, paraffin wax, phase change material

## Abstract

Paraffin wax stores energy in the form of latent heat at a nearly constant temperature during melting and releases this energy during solidification. This effect is used in industrial energy storage. At the same time, the possible deformation of even small volumes of material as a result of phase change is insufficiently studied. In this paper, the physical nature of such deformation, probably for the first time, is studied on the example of a droplet of paraffin wax. An unusual change in the shape of a melted droplet of paraffin wax placed on a relatively cold glass plate was observed in the laboratory experiments. As the droplet solidifies, its upper surface becomes nearly flat, and a dimple is formed in the center of this surface, making the droplet look like a fruit (pumpkins are more commonly shaped like this, but the authors prefer apples). A series of experiments, as well as physical and numerical modeling of the droplet’s thermal state, taking into account the formation of a mushy zone between liquidus and solidus, made it possible to understand the role of gravity and gradual increase in viscosity and density of paraffin wax on changing the droplet shape and, in particular, to clarify the mechanism of formation of the dimple on its upper. It was shown that the mushy zone between the liquidus and solidus of the paraffin wax is responsible for the dimple formation.

## 1. Introduction

It is known that solidification of the droplets leads to a surprising variety of shapes, ranging from the shapes of the metamorphic snowflakes [1,2] to the paradoxical pointed “freezing tip” observed for frozen water and germanium droplets [3,4,5,6,7]. The sharp tip registered during the cooling of water droplets exhibits a remarkable universality in its shape, independent of the cooling regime and physical properties of the substrate [4,5,6,7,8,9], which has been attributed to the jump in water density during freezing. Dendrite-like morphology has recently been observed on the outer shell of oil-coated frozen droplets [10]. The shape of the solidified wax droplets was studied intensively in view of their applications in 3D solid-inkjet printing [11,12]. It is usually accepted that wax droplets adopt spherical shapes under solidification [11,12].

In the present study, we investigated experimentally and theoretically the solidification of paraffin wax droplets. We observed a centrally concave, apple-like shape, which can be seen as the opposite of a freezing tip shape. Typically, centrally concave shapes are observed when water droplets impact cold solid surfaces [13,14,15,16]. The resulting shape is due to a combination of impact dynamics and the freezing process [13,14,15,16]. The formation of a central dimple is explained by the additional internal circular freezing front advancing both upwards and toward the periphery of the droplet [13]. Our experiments, in which a centrally concave shape of solidified paraffin wax droplets was observed, were almost static. Therefore, the observed shape of the solidified droplet is not related to the impact of the droplet on the solid plate. The centrally concave shape of solidified paraffin droplets was reported in ref. [17], in which it was noted that these shapes resemble those inherent to spreading oceanic ridges. Concave wax droplets formed at the water-air interface were reported in ref. [18]. 

As far as we know, the explanation of the formation of dimples on the upper surface of solidifying paraffin droplets is given in the present work for the first time. It should be noted that understanding the solidification process of paraffin wax is important for a better understanding of the thermal and mechanical behavior of this material, which is widely used in engineering applications, such as 3D printing and energy storage. Like other phase change materials (abbreviated PCM), paraffin wax stores energy in the form of latent heat at a nearly constant temperature during melting and releases this energy during solidification. This effect is used in industrial energy storage [19,20,21,22,23]. The latent thermal energy storage mechanisms used in PCM are attracting increasing attention for solar energy storage because of their environmental friendliness and sustainability. Paraffin wax is one of the most popular PCM materials used for energy storage. It was demonstrated recently that the presence of dimples at the surface of the droplet increases the heat transfer coefficient at a rate greater than can be attributed to the increasing surface; thus, the deformation of the surface may give rise to an essential thermal effect, which is of primary importance for the use of paraffin as a phase change material [22,23,24,25,26,27,28]. 

## 2. Materials and Methods

### 2.1. Materials

The materials used in the experiment were paraffin wax poly(dimethylsiloxane) (PDMS) Sylgard 184, supplied by Dow Corning, Midland, MI, USA. Some properties of the material are as follows: melting point 46 °C–50 °C, the specific heat capacity of the solid material—2.95 kJ/kg K, the specific heat capacity of the liquid material—2.51 kJ/(kg K), the density of the solid material—818 $kg/m^{3}$, the density of the liquid material—760 $kg/m^{3}$, the thermal conductivity of solid and liquid material—0.24 W/(m K).

### 2.2. Experimental Procedure

**Figure 1:**
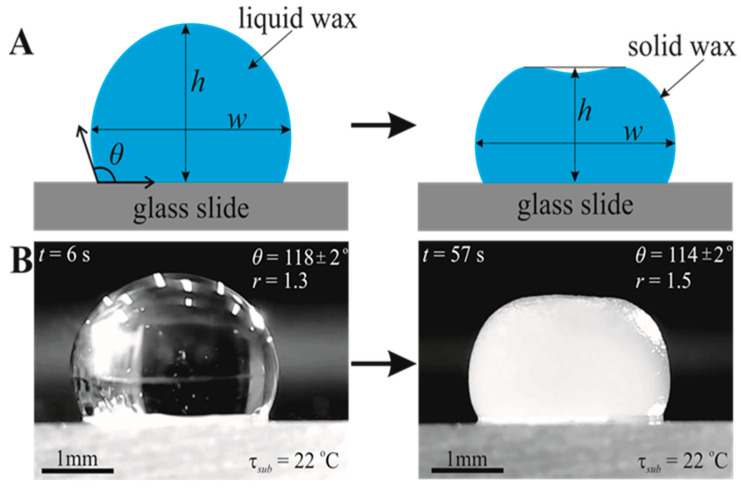
Solidification of molten paraffin wax. (**A**) Schematic representation of the solidification of a liquid paraffin wax droplet, where *θ* is the contact angle, and *h* and *w* are the height and maximum width of the droplet, respectively. (**B**) Images of a 10 µL droplet before and after solidification on a glass slide at $\tau_{\mathrm{sub}}=22\;{}^{\circ}C$, where aspect ratio is $r=\frac{w}{h}$ and τ is the glass slide temperature.

First, the solid paraffin wax beads were melted in a metal container at 150 °C. Then using a micro-syringe, liquid paraffin wax was placed on a glass slide, as shown in Figure 1. The glass slide was at room temperature (22 °C). The total time to place a droplet on a glass slide from the metal container is 5±2 s. Molten droplets were gently placed on the glass slide to minimize the droplet impact. 10 µL to 100 µL droplets were used to study the formation of the eventual shape of the droplet under its solidification. The change in the shape of the droplet is illustrated in Figure 1. The temperature of different positions of the wax droplets (50 µL and 100 µL) were measured using a TM-902C Lutron Digital Thermometer (Lutron, Coopersburg, PA, USA) with a lab-made chromel–alumel thermocouple with an error of ±1 °C as shown in Figure S4. The top surface temperature of 50 µL droplets was measured using a Therm-App Pro TAS19AV-M25A-HZ LWIR 7.5–14 μm infrared camera (Opgal Optronic Ind, Karmiel, Israel), as shown in Figure S5. The resolution, sensitivity, and frame rate of the camera were 640 × 480 pixels (>300,000 pixels), noise equivalent differential temperature (NEDT) < 0.03 °C, and 18 FPS, respectively.

The experiments were conducted at room temperature τ=22 °C and relative humidity 40–58%. The experimental freezing conditions corresponded to those inherent in 3D printing, and paraffin wax was used as a phase change material. To check the effect of gravity on the solidification of molten wax droplets, an experiment was performed on a rotating platform, as shown in Figure S3. A 10 µL molten paraffin wax droplet was placed on a glass slide and immediately rotated 180° about the horizontal axis so that the droplet was hanging vertically on the glass slide.

**Figure 2:**
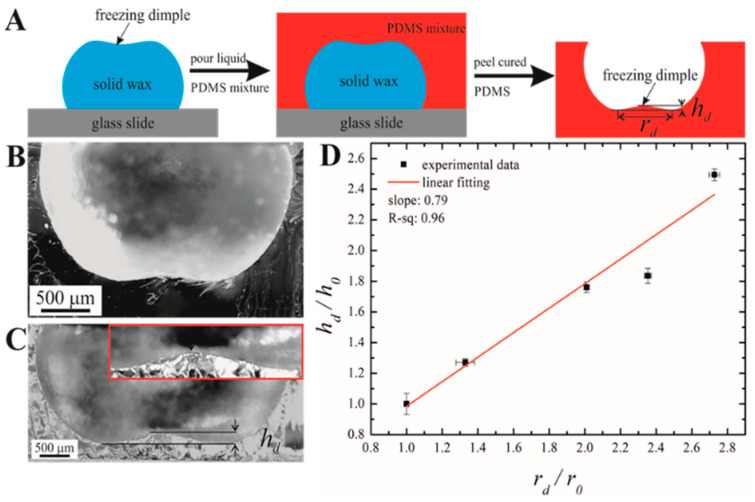
Replication and measurement of the solidified dimple. (**A**) Schematic representation of the replication process of the solid paraffin wax droplet using PDMS mixture (**B**,**C**) are the cross-section images of PDMS replicas of 10 µL and 50 µL droplets, respectively. (**D**) Variation of dimple depth with wax dimple radius where $r_{0}=0.42\pm 0.01\;mm$, and $h_{0}=85.8\pm 0.1\;\mu m$.

A replica of the solidified droplets was taken using PDMS to analyze the freezing dimple. A mixture of PDMS and its curing agent in a weight ratio of 10:1 was prepared for replication via stirring using a glass rod for 15 min and degassing in a low-pressure chamber for 30 min. After that, the liquid PDMS mixture was poured on the solidified droplet and cured the PDMS at ambient conditions for 5 days. After curing, solid PDMS was peeled from the wax droplet, as shown in Figure 2. A cross-section was taken from the cured PDMS to analyze the freezing dimple.

A digital microscope BW1008-500X was used to capture the solidification process. The optical resolution of the microscope was 640×480 pixels. Apparent contact angles (APCA), denoted *θ* were measured using ImageJ (National Institutes of Health, Bethesda, MD, USA) from the images captured using the digital microscope BW1008-500X (New Taipei City, Taiwan) with an accuracy Δθ=0.5°. Ten measurements were performed under ambient conditions; the results were averaged.

## 3. Results

### In Situ Study of the Shape of Heated and Cooled Paraffin Wax Droplets

Droplets (V=10–100 μL) were placed on the heated from below glass slides, melted, and solidified. The maximal temperature of the droplets was τ=150±1 °C. The eventual temperature of the droplets was τ=22±1 °C. The heating/solidification cycle was carried out over a period of time $t_{c}=t_{heat}+t_{solid}$; $t_{heat}≅10–20\;s;t_{solid}≅25–60\;s$, depending on the volume of the droplet. The droplet’s shape, which changed during the heating/solidification cycle, was constantly monitored. The droplet, the top surface of which was originally close to spherical, took the shape shown in Figure 1. Under the action of gravity, the droplet changed its shape even before it began to solidify (see Videos S1–S3 in Supplementary Materials). The shape of its upper surface became like an oblate spheroid (see refs. [29,30]). Solidified droplets of volume 10 µL and 20 µL have an axisymmetric dimple on their upper surfaces, as shown in Figure 1. 

The shape of the dimple was studied with the PDMS replica method (also called “soft lithography” [31]), illustrated in Figure 2 and described in detail in the Experimental Procedure section. Soft lithography enabled a detailed study of the dimple shape; in particular, the opening angle of the dimple, denoted *β*, was determined, as shown in Figure S1 (Supplementary Materials).

The initial apparent contact angle denoted θ for the paraffin/air system was established at ambient conditions as θ=118±2°. Note that the value of *θ* depends strongly on the temperature of the substrate. Thus, the entire process of heating and solidifying the droplets depends crucially on the initial temperature of the substrate, as illustrated in Figure S2. The apparent contact angle decreases with increasing substrate temperature; starting from substrate temperature $\tau_{sub}=60\;{}^{\circ}C,$ full wettability of the glass plate with paraffin wax was observed, as shown in Figure S2 (inset c).

Interestingly, the solidification processes for small and larger droplets are markedly different. In Video S1, for a 10 µL droplet, the solidifying lower layer of the droplet first becomes opaque. Then the zone of opacity (though not so pronounced) spreads from bottom to top, covering the whole lateral surface of the droplet. As shown in the computational section below, partial loss of transparency of paraffin occurs when the so-called mushy zone is formed, in which the paraffin wax is no longer liquid but also not completely solidified. In Video S2, a thin opaque layer of paraffin wax can be seen on a 20 µL droplet, appearing on the upper surface of the droplet while the middle part of the droplet is still transparent. This effect is much stronger with a 50 µL droplet (see Video S3), where the two areas of opacity in the lower and upper part of the droplet extend and meet in the middle of the droplet. This change of solidification pattern towards larger droplets is accompanied by the disappearance of a dimple on the upper surface already at 50 µL droplet volume. The below presented computational analysis of the unsteady temperature field in a solidifying droplet explains the physical cause of the observed phenomenon.

**Figure 3:**
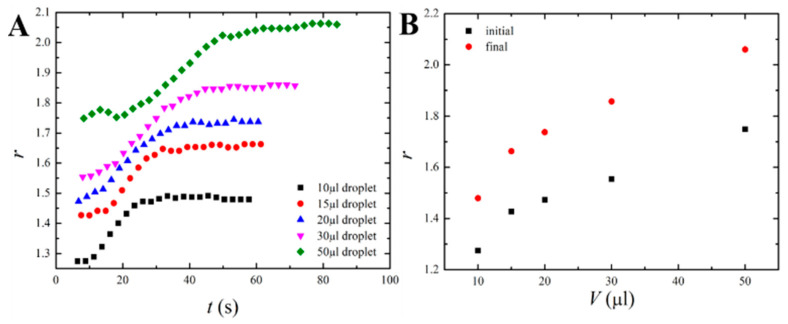
Change of the droplet’s shape with time. (**A**) Variation of aspect ratio (*r*) vs. time (*t*) for different paraffin wax droplets placed on a glass surface at τ_sub_ = 22 °C. (**B**) Variation of aspect ratio (*r*) vs. droplet volume (*V*).

To clarify the role of gravity, we also performed the experiments hanging upside down, as shown in Figure S3 and Video S4. In these experiments, the formation of the dimple on the upper surface of the droplet was not observed. As one might expect, the effect of gravity is more significant for large droplets: such droplets, placed on the upper surface of a glass plate, are compressed more strongly along the vertical axis. The flattening effect of gravity on the droplet shape leads to a small increase in the contact radius and a decrease in droplet height (as shown in refs. [29,30]), with these effects being more pronounced at larger values of the Bond number, which will be explicitly calculated below. The shape of the droplet was characterized by the aspect ratio, introduced according to Equation (1):

(1) $$r=\frac{w}{h},$$

where *h* and *w* are the height and maximum width of the droplet correspondingly, as shown in Figure 1. Time evolution r(t) for different droplet volumes is depicted in Figure 3. The aspect ratio of the droplets grew under their heating and came to saturation under their solidification (all of the droplets were heated to τ=150 °C and cooled to τ=22 °C).

**Figure 4:**
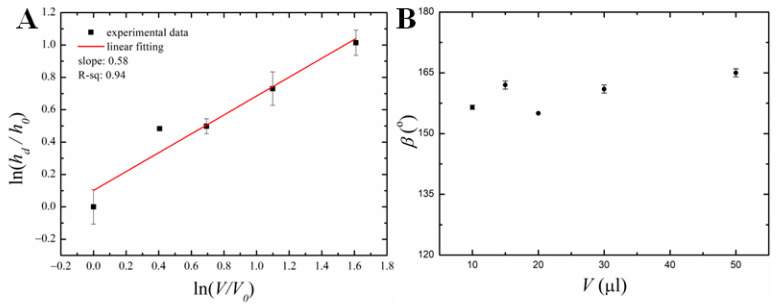
Variation of dimple depth and tip angle with volume. (**A**) Logarithmic plot of freezing dimple depth vs. different initial molten wax droplet volume, where *V*_0_ = 10 μL and *h*_0_ = 66 ± 5 μm. (**B**) Variation of tip angle with different molten wax droplet volumes.

The aspect ratio does not exhaust the geometrical characterization of the droplet shape; the double logarithmic dependence of the eventual value of the maximal dimple depth $h_{d}$ on the initial volume of the droplet is presented in Figure 4A. 

The double-logarithmic dependence is well approximated by the linear fitting:

(2) y=kx+b,

where $y=ln\frac{h_{d}}{h_{0}}$, $x=ln\frac{V}{V_{0}}$, $k=0.58,b=0.1,h_{0}=66\;\mu m,V_{0}=10\;\mu L.$ This means that the dimensionless dimple depth $\bar{h}=\frac{h_{d}}{h_{0}}$ and initial volume of the droplet ${\bar{V}}_{0}=\frac{V}{V_{0}}$ are related by the following scaling relationship:

(3) $$\bar{h}\sim {\bar{V}}_{0}^{0.58}$$

The physical basis for this dependence will be clarified in the last section of the paper.

**Figure 5:**
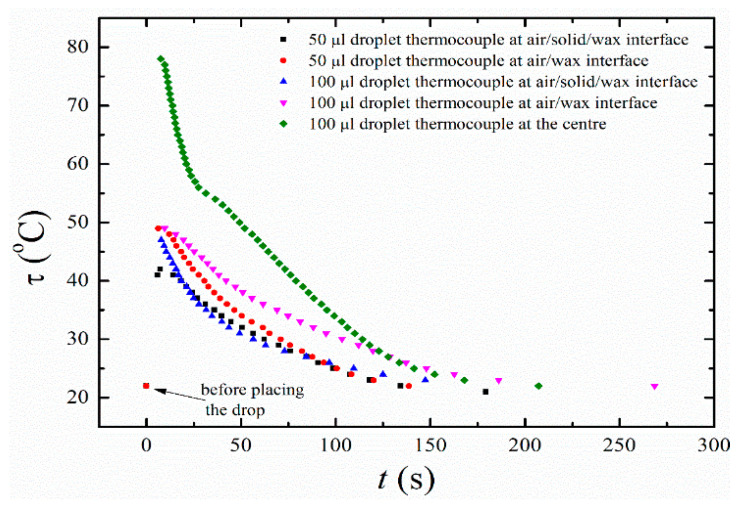
Variation of the surface temperature of paraffin wax droplets with time.

**Figure 6:**
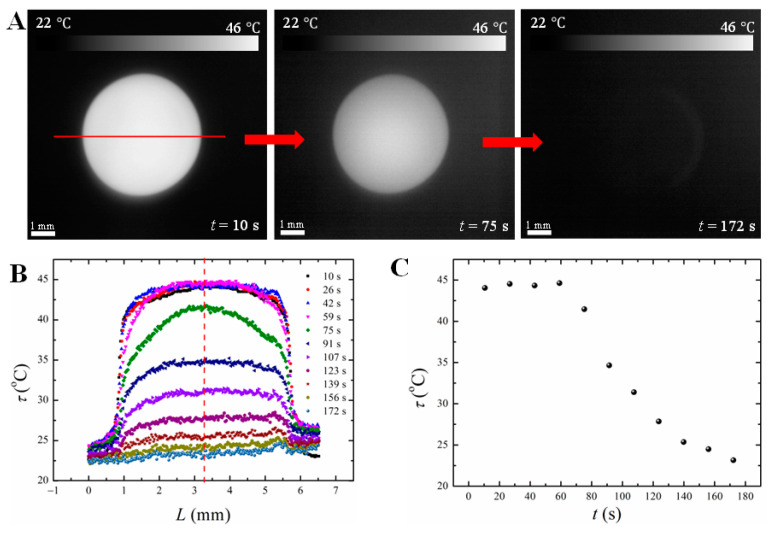
Variation of the brightness temperature of the droplet with time. (**A**) Thermal images (top view) of a 50 µL wax droplet at different times. (**B**) Temperature profile of a 50 µL wax droplet. The red line inset (**A**) is the scan line. (**C**) Temperature variation with time at the center point of the droplet top surface (the red dash line shown in inset (**B**) represents the center point).

The temperature of the substrate and paraffin wax droplet was monitored as described in detail in the Experimental Procedure section. The time-varying temperature of the bottom and side of the droplet was measured with a thermocouple (see Figure 5 and Figure S4), while infrared pyrometric measurements were used for the temperature of the top surface of the droplet (see Figure 6 and Figure S5, and Video S5). The results of these temperature measurements are used below in the computational analysis of the transient temperature field in the paraffin wax droplet.

## 4. Discussion

### 4.1. Qualitative Analysis

Let us start with dimensionless numbers relevant to the reported experiments. The Bond number, describing the interrelation between the gravity and surface tension-inspired effects:

(4) $$Bo=\frac{\rho gd^{2}}{\gamma },$$

where γ and ρ are the surface tension and density and paraffin wax, respectively, *g* is the acceleration of gravity, and *d* is the characteristic dimension of the droplet. Assuming for the sake of a rough estimation $≅8.0\times 10^{2}\frac{kg}{m^{3}}$, $\gamma ≅20.0\times 10^{-3}\frac{J}{m^{2}}$ (see ref. [32]) and d≅1.0–2.0 mm yields Bo=0.8–3.2. This means that the effects due to gravity and surface tension are comparable. The Reynolds number is given, in turn, in Equation (5):

(5) $$Re=\frac{\rho vd}{\eta },$$

where *v* is the characteristic velocity of the paraffin flow during the droplet deformation, η is the dynamic viscosity. The dynamic viscosity of paraffin wax is varied in a very broad range, namely $\eta \left(\tau =29\;{}^{\circ}C\right)≅10^{10}\;Pa\times s;\eta \left(\tau =52\;{}^{\circ}C\right)≅10\;Pa\times s$ (see ref. [33]). The characteristic velocity was estimated experimentally as $v≅10^{-5}\frac{m}{s}.$ Substitution of the aforementioned values of physical parameters yields $Re<10^{-6}.$ This means that inertial effects are negligible in the deformation of the paraffin wax droplet.

### 4.2. A Model of Cooling and Solidification of a Paraffin Wax Droplet

The above observations of the changing shape and the gradual solidification of the paraffin wax droplet show that these processes are interrelated in a rather complex way. Nevertheless, an initial relatively rapid deformation of the droplet by gravity, when the droplet is not yet cooled, can be distinguished. Experiments have shown that the deformation of the droplet continues throughout the cooling and solidification of the droplet, but this deformation is much slower.

When the lower layer of the droplet solidifies and a solid-liquid mushy zone is formed in the entire surface layer of the droplet (see Videos S1–S3 in Supplementary Materials), the surface tension can no longer hold the same shape of the droplet and the height of the droplet begins to decrease considerably. A similar effect was observed when cooling from the bottom of water marbles covered with a dense layer of fine powder (see ref. [34]) but with a surface ice layer instead of the mushy zone. Of course, a droplet of higher-viscosity paraffin wax deforms much more slowly. The exponential increase in viscosity of liquid paraffin wax with decreasing temperature (this can be found in ref. [35]) makes it difficult to estimate the rate of deformation using the ratio $\nu /\left(Rg\right)$ for the characteristic time of the process (here ν is the kinematic viscosity of the material, R is the radius of an initially nearly spherical droplet, g is the acceleration of gravity). It should be noted that a slow decrease in droplet height continues for some time during the droplet solidification.

**Figure 7:**
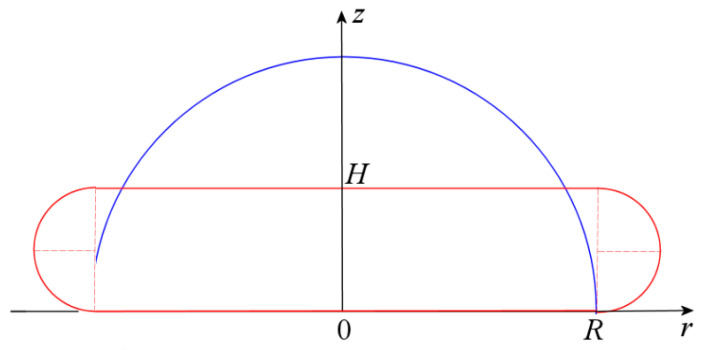
Schematic of the shape of the paraffin wax droplet: blue curve—initial shape, red curve—final shape.

The slight increase in the density of paraffin wax during its solidification (only 9%) simplifies the estimation of the height of the solidified droplet. We will assume an approximate representation of the initial shape of the droplet as a hemisphere and assume that the top surface of the solidified droplet is flat (see Figure 7).

Assuming the material density remains unchanged, determine the height of the compressed droplet from the equality of the initial and final volumes with Equation (6):

(6) $$\frac{2}{3}\pi R^{3}=\pi R^{2}H+\pi {\left(\frac{H}{2}\right)}^{2}\times 2\pi R$$

Equation (6) gives the following formula for the relative height of the compressed droplet:

(7) $$\bar{H}=\frac{H}{R}=\frac{2}{\pi }\left(\sqrt{1+2\pi /3}-1\right)\approx 0.48$$

Taking into account the increase in the material density with solidification, we obtain $\bar{H}\approx 0.44$. This value is slightly less than that in the experiment, but it is not important for an approximate computational model.

Since the formation of a dimple in the center of the upper surface of a solidified droplet is of the main physical interest, we will focus on solving the problem of cooling and solidifying the droplet. It is natural to assume that the dimple formed before the full solidification of the droplet is due to a small volume of liquid paraffin wax remaining under the more viscous part of the mushy zone near the upper surface of the droplet. Volume reduction during the solidification of viscous paraffin wax in this area causes the surface layer to subside. To check this assumption, we should solve a 2D axisymmetric problem of transient heat conduction considering the latent heat of phase transition.

Considering the difficulty of quantifying the change in droplet shape when solving the thermal problem, we restrict ourselves to a simpler calculation, assuming that the droplet shape remains unchanged and is the same as the final shape. This relatively simple model is acceptable due to the very low thermal conductivity of paraffin wax. This value for liquid paraffin wax is 3.4 times lower than that of water, and the thermal conductivity of solid paraffin wax is 6.4 times lower than that of ice. Therefore, when cooling from below, the temperature field in most of the droplet volume is almost independent of the deformation of the upper surface of the droplet.

The temperature field in a droplet is determined as a solution to a transient heat transfer problem in the axisymmetric computational region with the cross-section shown in red in Figure 7. The axial coordinate z is measured from the glass plate, and the contact area of the droplet with the plate is a circle of radius R. The conduction equation for the temperature field $\tau \left(t,r,z\right)$ is as follows:

(8) $$\rho c\frac{\partial \tau }{\partial t}=\frac{1}{r}\left(kr\frac{\partial \tau }{\partial r}\right)+\frac{\partial }{\partial z}\left(k\frac{\partial \tau }{\partial z}\right)$$

where ρ, c, and k are the density, specific heat capacity, and thermal conductivity of paraffin wax, respectively. To take into account the latent heat $L_{m}$ of phase change for substances whose phase change is characterized by the melting/solidification point $\tau =\tau_{m}$ one can use an equivalent additional capacity in the temperature range of $\tau_{m}-\Delta \tau <\tau <\tau_{m}+\Delta \tau$, where $\Delta \tau \ll \tau_{m}$, as it was done in refs. [36,37,38]:

(9) $$\Delta c=\frac{L_{m}}{\Delta T}\left(1-\frac{\left|\tau_{m}-\tau \right|}{\Delta \tau }\right)$$

When paraffin wax solidifies, a mushy zone is formed in the region of the temperature between the liquidus and solidus (see refs. [39,40,41]): $\tau_{sol}<\tau <\tau_{liq}$. In our case, the values of $\tau_{m}$ and Δτ in Equation (9) should be replaced by $\left(\tau_{liq}+\tau_{sol}\right)/2$ and $\left(\tau_{liq}-\tau_{sol}\right)/2$, respectively. We will also assume that the density, specific heat capacity, and thermal conductivity depend linearly on the temperature in the mushy zone. In the case of paraffin wax, the volume occupied by the mushy zone should be comparable to the droplet volume because of the very low thermal conductivity of the material and the significant difference between the liquidus and solidus temperatures. This expectation is confirmed by the numerical results presented below.

The initial condition and the boundary conditions for Equation (8) are:

(10) $$\tau \left(0,r,z\right)=\tau_{0}$$

$$\frac{\partial \tau }{\partial r}=0\;\mathrm{at}\;r=0;\;\tau =\tau_{g}\left(t\right)\;\mathrm{at}\;z=0;$$

(11) $$\tau =\tau_{a}\left(t\right)\mathrm{on}\mathrm{the}\mathrm{surfaces}$$

where $\tau_{g}\left(t\right)$ and $\tau_{a}\left(t\right)$ are the measured surface temperature of the droplet at the contact zone with the glass plate and at the surface surrounded by air. According to infrared pyrometric measurements of the brightness temperature of paraffin wax droplets, we assume that the temperature of the upper surface of the droplet is independent of the radial coordinate.

**Figure 8:**
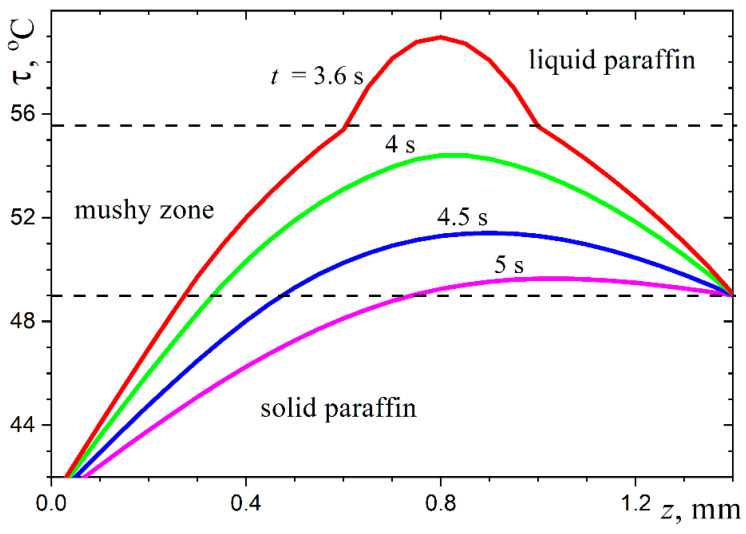
Temperature profiles along the vertical axis of the droplet.

**Figure 9:**
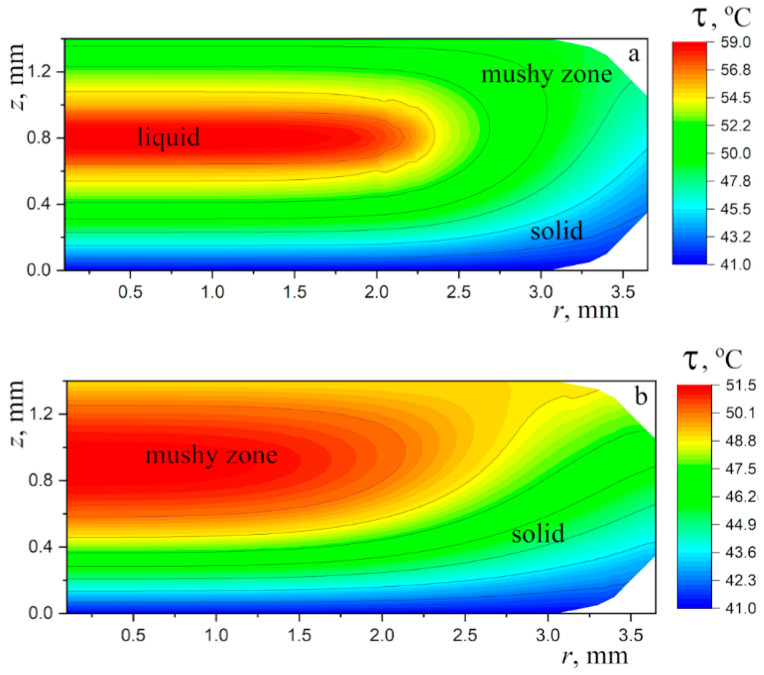
The temperature field in the paraffin wax droplet: (**a**) t=3.6 s, (**b**) t=4.5 s. The mushy zone is between the isotherms τ=49 °C and 56 °C.

The above heat conduction problem was solved numerically using an implicit finite-difference scheme of the second order of approximation on an orthogonal grid, with splitting the operator on the right side of Equation (8), as done in refs. [42,43]. The thermophysical properties of the different types of paraffin wax are essentially different (see refs. [44,45,46]). The following values have been assumed for the calculations: $\rho_{\mathrm{liq}}=790$ kg/m^3^, $\rho_{\mathrm{sol}}=916$ kg/m^3^, $c_{\mathrm{liq}}=3.26$ kJ/(kg K), $c_{\mathrm{sol}}=1.92$ kJ/(kg K), $k_{\mathrm{liq}}=0.167$ W/(m K), $k_{\mathrm{sol}}=0.346$ W/(m K), $\tau_{\mathrm{liq}}=329$ K, $\tau_{\mathrm{sol}}=322$ K, and $L_{m}=174$ kJ/kg. The results of the temperature field calculations for a typical paraffin wax droplet are presented in Figure 8 and Figure 9.

It can be seen that by the time the lower layer, about a quarter of the droplet height, has solidified, the rest of the droplet volume is occupied by the mushy medium. This can be seen as a confirmation of the supposition made above, based on the specific thermal properties of the material. As a result, the hottest zone, dominated by liquid paraffin wax, has the appearance of a lens close to the upper surface of the droplet. This confirms the general assumption made above about the physical nature of the dimple on the upper surface of the paraffin wax droplet.

Obviously, the relative width of the hot lens below the upper surface of the droplet depends on the droplet size. In very small droplets, this zone cannot be as wide and almost flat as in Figure 9, and in very large droplets, the hot lens, on the contrary, is almost flat, and its width is only a little less than the diameter of the upper surface of the droplet. In both cases, there are no conditions for the formation of a dimple on the upper surface. A derivation of the two-dimensional heat transfer problem for large droplets where the temperature field in most of the droplet volume can be calculated by solving a one-dimensional problem considering heat transfer only in the axial direction is an extreme case showing that the appearance of a non-wide hot lens and the resulting formation of a dimple is possible only for small (but not too small) droplets. This conclusion, based on considering the heat transfer parameters of the paraffin wax droplet, explains the observed change in the solidification pattern of the droplet as its size changes (see Videos S1–S3). The suggested approximate computational model does not give accurate temperature values in each element of the droplet volume at each point in time for several obvious reasons. One source of systematic error is the simplified representation of the shape of the computational region; another is the natural uncertainty in the values of the thermo-physical parameters, which in addition, are different for different types of paraffin. However, this by no means prevents one from obtaining a physically correct picture of the thermal process and correctly understanding the solidification features of paraffin wax droplets and other phase-change materials.

The above computational analysis enables us to understand the dimple formation on the upper surface of the solidified droplet. This process stage begins when the paraffin wax on the droplet’s surface is close to solidification, and there is a hot mushy zone with high liquid paraffin wax content below it, near the axis of the droplet. Continued cooling causes the volume of the initial liquid lens to decrease, resulting in a thin crust of paraffin wax descending and forming a dimple on the surface of the solidified droplet. The results of numerical modeling agree well with the laboratory observations. One can see in Videos S1 and S2 from the Supplementary Materials that, at first, only the solidified thin bottom layer of the droplet becomes white (opaque); then the image of the rest of the droplet of paraffin wax gradually becomes turbid (when the mushy zone extends to the whole volume of the droplet), and finally, the dimple of interest appears on the top surface of the droplet. The authors consider the above physical analysis to be the novelty of the present study.

Theoretical estimation of the final dimple depth can be based on the following simple considerations. First, the measurements demonstrate that the growth of the dimple preserves the self-similarity to a large extent since the aspect ratio and the angle are volume-independent (cf. Figure 2D and Figure 4B). Then, assuming the shape of the dimple as a spherical segment, its volume is expressed as follows:

(12) $$V_{d}=\frac{\pi h_{d}^{3}}{3}\frac{2+\sin(\beta /2)}{1-\sin(\beta /2)}$$

Then, we assume that the dimple grows in accordance with the dynamics of the bulk volume relaxation in the course of solidification. We assume that this process is characterized by some characteristic time $t_{r}$ for the bulk, and only the part of the volume that is “relaxed out” actively participates in the process. Thus, the following empiric estimation is in order:

(13) $$\frac{dV_{d}}{dt}\approx \frac{\rho_{sol}-\rho_{liq}}{t_{r}\rho_{sol}}V$$

The process of dimple growth stops when the droplet solidifies; we assume that it happens after some characteristic time $t_{f}$. In all cases, the volume of the dimple is much smaller than the volume of the droplet, and it is possible to estimate:

(14) $$V_{d}\sim \frac{\rho_{sol}-\rho_{liq}}{t_{r}\rho_{sol}}Vt_{f}$$

The characteristic solidification time depends on the size of the droplet and the thermal conductivity:

(15) $$t_{f}\sim \frac{\rho cR^{2}}{k}∼\frac{\rho cV^{2/3}}{k}$$

The indices related to the solid or liquid phase in Equation (15) are omitted since, at this level of qualitative estimation, there is no need to distinguish between them. Also, there is no need to distinguish between the running droplet volume *V* and the initial volume $V_{0}$. With the account of Equations (12), (14), and (15), one arrives at the following estimation for the dimple height:

(16) $$h_{d}\sim {\left(\frac{1-\sin(\beta /2)}{2+\sin(\beta /2)}\frac{\rho_{sol}-\rho_{liq}}{t_{r}\rho_{sol}}\frac{c\rho }{k}\right)}^{1/3}V_{0}^{5/9}$$

The exponent in Equation (16) agrees with numeric estimation (3). If we substitute the measured value of the angle β≈160°, average values for the heat capacity, density, and heat conductivity, and assume $t_{r}\sim 100$ s in accordance with our experimental observations (cf. Figure 5), then for the droplet volume $V_{0}=50$ µL one obtains $h_{d}\sim 340$ µm, which is in reasonable qualitative agreement with the experimental findings.

## 5. Conclusions

Paraffin wax is a phase change material, which stores energy in the form of latent heat at a nearly constant temperature during melting and releases this energy during solidification. This effect is used in industrial energy storage. It is also interesting to consider the possible deformation of such material at volumetric phase changes. In this work, just such an unusual deformation of a paraffin drop, resulting in the eventual “apple-like” shape was, perhaps for the first time, not only observed but also physically investigated. An experimental and theoretical study of this deformation of droplets placed on a relatively cold glass plate was carried out. Deformation of the droplet by gravity begins before the droplet cooling and continues, but at a much slower rate, as the droplet cools and solidifies. At the final stage of the process, a dimple is formed on the upper surface of the droplet. The thermal mechanism of the formation of this dimple has been the main subject of our analysis.

In laboratory experiments, video recording of the droplet was used, which not only showed different stages of the droplet deformation but also, due to the semitransparency of liquid paraffin, allowed to observe solidification of the lower part of the droplet and formation of the mushy zone between the liquidus and solidus of paraffin wax in the droplet. The temperature measurements were used to complete the statement of the transient heat conduction problem for the paraffin wax problem. The numerical solution has taken into account the latent heat of the phase change and variable thermal properties of the material in the mushy zone. The calculations showed that a relatively hot “lens” formed in a small droplet moves towards the central part of its upper surface. The solidification of the paraffin wax in the lens causes its volume to decrease, and a thin surface layer of nearly solid material descends to form the central dimple. Heat transfer analysis has shown that such a dimple cannot be formed on very small or too-large droplets of paraffin wax. The latter is confirmed by the observed solidification patterns of droplets of different sizes. The physical explanation of the formation of dimples on the upper surface of solidifying paraffin droplets is given in the present work for the first time. Our analysis is expected to be important for a better understanding of the thermal and mechanical behavior of the phase change materials. The reported results are also important for developing 3D printing processes, exploiting paraffin wax droplets. 

## Data Availability

The data supporting the findings of this study are available from the corresponding author upon reasonable request.

## Supplementary Materials

The following supporting information can be downloaded at https://www.mdpi.com/article/10.3390/ma16165514/s1: Figure S1: Opening angle; Figure S2: Effect of substrate temperature on molten wax wetting behavior; Figure S3: Effect of gravity on droplet freezing; Figure S4: Thermocouple measurements of the droplet surface temperature; Figure S5: Schematics of pyrometric temperature measurements; Video S1: Side view of a deforming and solidifying 10 µL paraffin wax droplet; Video S2: Side view of a deforming and solidifying 20 µL paraffin wax droplet; Video S3: Side view of a deforming and solidifying 50 µL paraffin wax droplet; Video S4: Side view of a deforming and solidifying 10 µL paraffin wax droplet hanging upside down from a glass substrate; Video S5: Time variation of infrared image of the paraffin wax droplet: 50 µL droplet, view from above, speed 7X.
