# Supplementary material for: Apple-like Shape of Freezing Paraffin Wax Droplets and Its Origin
Source: Materials (Basel). 2023 Aug 8;16(16):5514. doi: 10.3390/ma16165514 (PMC10456291; doi:10.3390/ma16165514)
Supplement: Supplementary file 1 [file materials-16-05514-s001.zip › Supplementary Materials.pdf]

materials

MDPI

Supplementary Materials

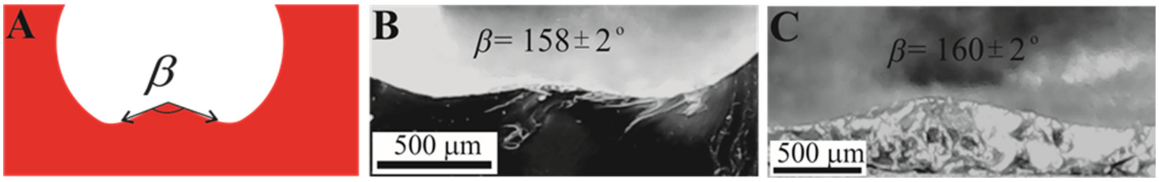

Figure S1. Opening angle. Images of the opening dimple angle (A) schematic, (B) replica taken from 10 μl paraffin wax droplet, (C) replica taken from 50 μl droplet.

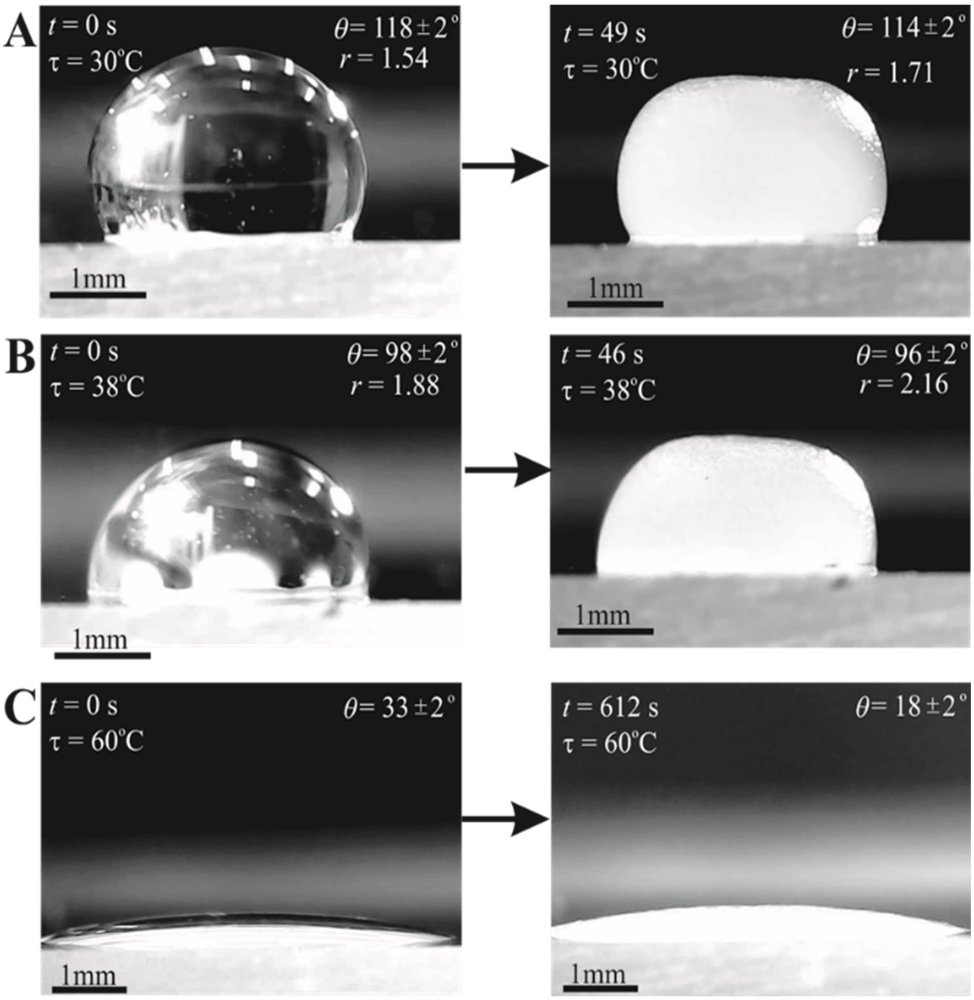

Figure S2. Effect of substrate temperature on molten wax wetting behavior. Images of 10 μl paraffin wax droplets placed on a slide at different temperatures: (A) 30 ℃, (B) 38 ℃, (C) 60 ℃.

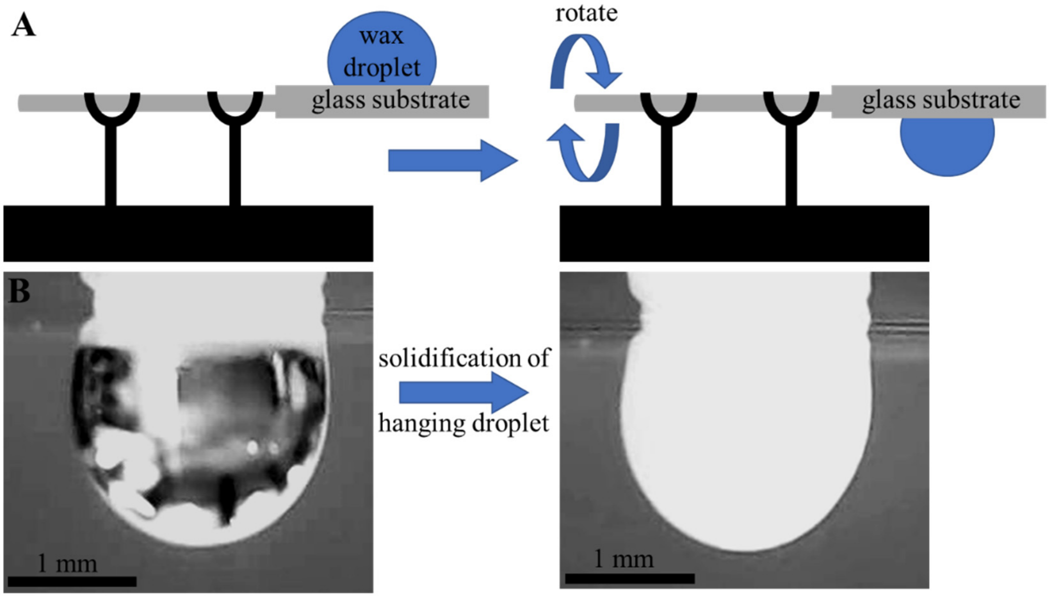

Figure S3. *Effect of gravity on droplet freezing. (A) Schematic representation of the experimental setup. (B) Images of a 10 µl paraffin wax droplet before and after solidification hanging from a glass substrate at* τ_{sub}*=22 ℃.*

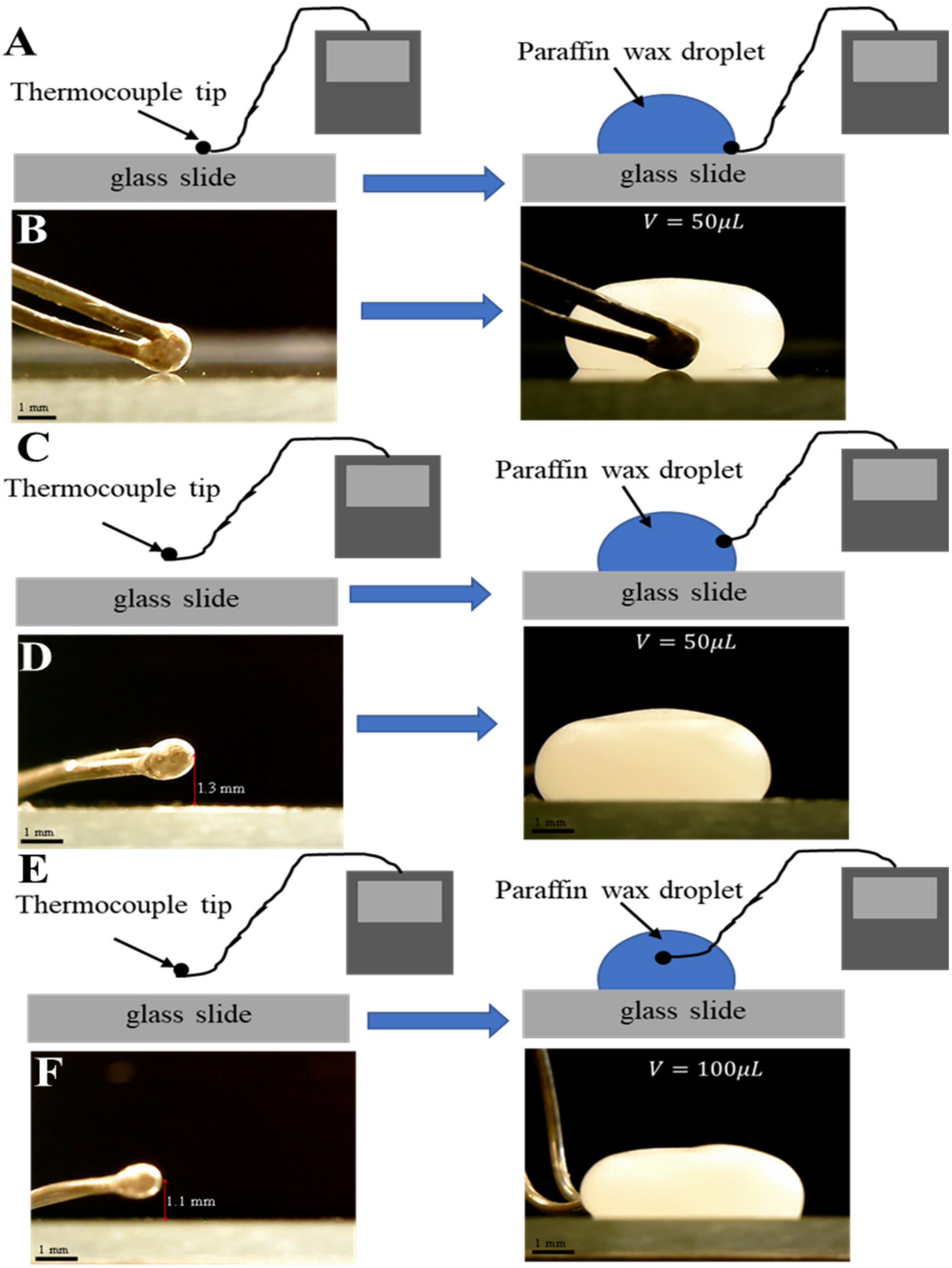

Figure S4. *Thermocouple measurements of the droplet surface temperature. Schematic (A) and image (B) represent the thermocouple tip placed at the three-phase contact line (air/glass/paraffin). Schematic (C) and image (D) represent the thermocouple tip at the air/paraffin interface. Schematic (**E**) and image (**F**) show that the thermocouple tip is surrounded by paraffin wax (near the center of the droplet).*

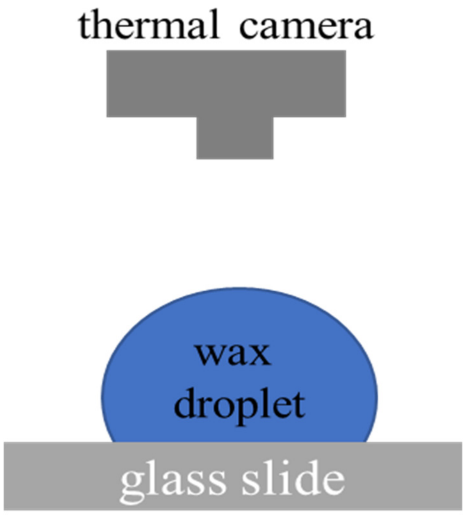

Figure S5. *Schematics of pyrometric temperature measurements.*

Supplementary Files

- **Video S1.** Side view of a deforming and solidifying 10 µl paraffin wax droplet.
- **Video S2**. Side view of a deforming and solidifying 20 µl paraffin wax droplet.
- **Video S3**. Side view of a deforming and solidifying 50 µl paraffin wax droplet.
- **Video S4**. Side view of a deforming and solidifying 10 µl paraffin wax droplet hanging upside down from a glass substrate.
- **Video S5**. Time variation of infrared image of the paraffin wax droplet. 50 µl droplet, view from above, speed 7X.
